# A Comparative Analysis of the Effects of Haloperidol and Dexmedetomidine on QTc Interval Prolongation during Delirium Treatment in Intensive Care Units

**DOI:** 10.2478/jccm-2024-0027

**Published:** 2024-07-31

**Authors:** Ali Haspolat, Fuat Polat, Ali Şefik Köprülü

**Affiliations:** Kolan International Hospital, İstanbul, Turkey; Dr Siyami Ersek Thoracic and Cardiovascular Surgery Training and Research Hospital, Istanbul, Turkey; Yeni Yuzyil University, İstanbul, Turkey

**Keywords:** delirium, QTc interval, haloperidol, dexmedetomidine, intensive care unit

## Abstract

**Background:**

Haloperidol and dexmedetomidine are used to treat delirium in the intensive care unit (ICU). The effects of these drugs on the corrected QT (QTc) interval have not been compared before. It was aimed to compare the effects of haloperidol and dexmedetomidine treatment on QTc intervals in patients who developed delirium during ICU follow-up.

**Method:**

The study is single-center, randomized, and prospective. Half of the patients diagnosed with delirium in the ICU were treated with haloperidol and the other half with dexmedetomidine. The QTc interval was measured in the treatment groups before and after drug treatment. The study’s primary endpoints were maximal QT and QTc interval changes after drug administration.

**Results:**

90 patients were included in the study, the mean age was 75.2±12.9 years, and half were women. The mean time to delirium was 142+173.8 hours, and 53.3% of the patients died during their ICU follow-up. The most common reason for hospitalization in the ICU was sepsis (%37.8.). There was no significant change in QT and QTc interval after dexmedetomidine treatment (QT: 360.5±81.7, 352.0±67.0, p= 0.491; QTc: 409.4±63.1, 409.8±49.7, p=0.974). There was a significant increase in both QT and QTc interval after haloperidol treatment (QT: 363.2±51.1, 384.6±59.2, p=0.028; QTc: 409.4±50.9, 427.3±45.9, p=0.020).

**Conclusions:**

Based on the results obtained from the study, it can be concluded that the administration of haloperidol was associated with a significant increase in QT and QTc interval. In contrast, the administration of dexmedetomidine did not cause a significant change in QT and QTc interval.

## Introduction

Research evidence suggests that delirium frequently develops in patients hospitalised in intensive care units (ICU), albeit with different degrees of severity [1]. Several factors may contribute to the development of delirium in ICU patients, including sedative drug use, mechanical ventilation, sleep disturbances, social context, and the presence of underlying medical conditions [2,3,4,5,6,7,8,9]. Sleep disturbances, such as disruption of normal circadian rhythms due to constant lighting and noise, are recognized as significant risk factors for delirium in the ICU [5,6]. Furthermore, the social environment, including isolation, lack of familiar faces, and limited interaction, can exacerbate delirium in critically ill patients [7,8]. Given the significant impact that delirium can have on patient outcomes, it is important for healthcare providers to closely monitor patients in the ICU and take proactive steps to prevent, identify, and treat delirium as early as possible [1]. Delirium is associated with severe consequences such as prolonged hospital and ICU stay, increased mortality, delayed weaning from mechanical ventilation, and nosocomial infections [2,3,4]. Haloperidol and dexmedetomidine are two drugs commonly used for sedation and delirium in the ICU [5,6,7,8,9,10,11].

Non-pharmacological pre-emptive approaches are increasingly recognized as vital in addressing cognitive disorders among ICU patients. Early mobilization, optimizing sleep hygiene, providing orientation cues, facilitating communication, and promoting social interaction have demonstrated efficacy in reducing the incidence and severity of delirium and other cognitive impairments in the ICU setting [1,2,3,4,5]. These interventions are essential as they address underlying factors such as sleep disturbances and social isolation, which are significant risk factors for cognitive decline in critically ill individuals.

In addition to these non-pharmacological approaches, various pharmacological compounds can also be used as treatment options for managing cognitive disorders in the ICU. Risperidone, for instance, is a commonly used antipsychotic medication that has gained popularity over haloperidol due to its favorable side effect profile. Other pharmacological options include benzodiazepines, alpha-2 agonists, and newer agents targeting specific neurotransmitter pathways implicated in delirium and cognitive dysfunction. Combining these pharmacological interventions with non-pharmacological strategies can lead to improved patient outcomes and overall quality of care in the ICU.

Haloperidol acts mainly by blocking dopamine receptors. Alpha-adrenergic and muscarinic receptors are partially blocked by haloperidol. The main indications for haloperidol are psychotic disorders, agitation, hallucinations, and delirium [12]. The most common adverse events associated with the cardiovascular system are electrocardiogram (ECG) changes, hypotension, tachycardia, and hypertension [13]. Several instances of torsades de pointes (TdP) or prolonged QT intervals associated with haloperidol have been reported [14,15].

Dexmedetomidine is a highly specific α_2_-adrenoceptor agonist with anxiolytic and analgesic effects. Dexmedetomidine effectively prevents delirium by acting on gamma-Aminobutyric Acid (GABA) receptors. Dexmedetomidine can effectively reduce delirium by creating a sedative and normal state of normal sleep in individuals [16,17,18]. The most common side effects of dexmedetomidine include hypotension, hypertension, nausea, bradycardia, atrial fibrillation, and hypoxia [19]. Although dexmedetomidine is on the list of drugs at risk of prolonging the QT Interval and inducing TdP, the available evidence for dexmedetomidine-induced QT prolongation is conflicting [20].

QT prolongation is an independent risk factor for the development of arrhythmias such as TdP, which lead to sudden cardiac death [21]. The effects of haloperidol and dexmedetomidine treatments, frequently used in the ICU, on the QT interval are contradictory. However, no prospective study has been conducted comparing the effect of the two drugs on QT prolongation. The primary objective of this study is to compare the effects of haloperidol and dexmedetomidine on QT prolongation in patients followed in the ICU. Secondary objectives include assessing the impact of these medications on clinical parameters such as systolic blood pressure, heart rate, and sedation scales.

## Methods

This study was a prospective, single-center, randomized trial to evaluate the efficacy of delirium treatment in a 65-bed intensive care unit. The study protocol was approved by the Local Ethics Committee meeting dated 17/05/2017 and numbered 2017/04 (IRB protocol No. 69396709-300.00.00-1001-1).

The study recruited 126 patients who developed delirium during their follow-up in the intensive care unit between June 2017 and July 2018. Prior to admission to the intensive care unit, explicit and documented informed consent was acquired from either the patient or their duly authorized legal representative. This informed consent encompassed the administration of pharmacological interventions for delirium and sedation, along with the attendant potential adverse reactions. It should be noted that the issue of obtaining informed consent for patients with delirium is a complex and sensitive matter. We took great care to ensure that all patients or their legally authorized representatives were fully informed about the study and that their consent was obtained in accordance with local regulations and ethical standards. Subsequent to the initial screening phase, consent was retracted by a total of eight patients, while an additional 28 patients were unable to fulfill the prescribed regimen for delirium interventions due to diverse factors. These factors encompassed pre-existing electrocardiographic (ECG) aberrations (n=8), concurrent administration of antiarrhythmic agents (n=6), deviant serum electrolyte levels (n=5), manifestations of allergic reaction or heightened sensitivity to α_2_-adrenergic agonists (n=3), as well as underlying neurological or psychiatric impairments (n=6). A total of 90 patients were enrolled in the study.

Patients were randomized to either the haloperidol (45) or dexmedetomidine group (n = 45) according to computer-generated random numbers (http://www.random.org). A 12-lead ECG was taken just before the start of delirium treatment and after the treatment was terminated. In ECG, the QT distance was measured by a cardiologist who did not know the patient’s assigned group. The QT intervals were measured manually by using the cursor from the earliest onset of the QRS complex to the latest end of the T wave, where its terminal limb joined the baseline. QT intervals were measured from lead V5, and the average of 4 consecutive beat measurements was recorded as the QT interval. Then, the QTc interval was calculated according to the Bazett formula [QTc= QT(ms)/RR(s)1/2]. Systolic blood pressure (SBP), heart rate, pulse oxygen saturation, Ramsay Sedation Scale, and Richmond Agitation-Sedation Scale (RASS) were recorded before and after drug administration. Height, weight, Acute Physiology and Chronic Health Evaluation (APACHE II) score, and Glasgow Coma Scale (GCS) were measured on the first day of the patient’s ICU.

Patients were excluded if they met any of the following criteria: pre-treatment ECG abnormalities, including a QTc interval of >450 milliseconds (ms), atrioventricular block, frequent premature atrial or ventricular complex; use of antiarrhythmic medications or other agents that are known to prolong the QTc interval; abnormal levels of serum electrolytes; any allergy or hypersensitivity to α_2_-adrenergic agonists; and neurological or psychiatric impairment. In addition, patients under 65 years of age were not included in the study because there was a warning against the use of dexmedetomidine in patients under 65 years of age. Delirium was diagnosed using the Confusion Assessment Method for the ICU (CAM-ICU). Patients who developed delirium during their follow-up in the 65-bed intensive care unit in a single center were included in the study. The occurrence of the different types of delirium was not specifically tracked in this study.

The primary focus of this study was to assess the efficacy of dexmedetomidine and haloperidol in treating delirium, irrespective of subtype. While it’s acknowledged that different subtypes of delirium may necessitate varied treatment approaches, this study primarily evaluated evidence-based main treatment modalities, including antipsychotics like haloperidol and alpha-2 agonists like dexmedetomidine. Non-pharmacological interventions such as reorientation, cognitive stimulation, and sleep promotion are also known to be effective in treating delirium, but they were not specifically evaluated in this study. Dexmedetomidine was administered with a loading dose of 1.0 microgram/kilogram (μg/kg), followed by intravenous infusion at a dose range of 0.2 to 1.4 μg/kg/hour (hr), adjusted at the clinician’s discretion to achieve an adequate efficacy dose. Haloperidol was given as a loading dose of 2.5 milligrams (mg), followed by intravenous infusion ranging from 0.5 to 2 mg/hr, with a maximum limit of 20 mg.

Maximum QT and QTc interval were assessed within 24 hours after drug administration. Post-treatment QTc interval over 450 ms and increase in QTc interval over 20 ms were compared between both groups. The effect of demographic and clinical patient characteristics on QTc interval was analyzed. Additionally, changes in SBP, heart rate, pulse oximetry oxygen saturation (SpO2), Ramsay sedation scale and RASS were compared after haloperidol and dexmedetomidine treatment.

The study objectives encompass comparing the effects of haloperidol and dexmedetomidine on QT prolongation in ICU patients as the main objective, while also evaluating the impact of these medications on secondary clinical parameters such as systolic blood pressure, heart rate, Ramsay Sedation Scale, and Richmond Agitation-Sedation Scale. Additionally, the study aims to analyze the correlation between demographic and clinical characteristics with QTc interval prolongation following medication infusion as other objectives.

### Statistical analysis

Patient baseline characteristics were compared with the χ^2^ test or Fisher exact test for categorical variables. Independent Samples T-test was used to compare the data of two normally distributed independent groups, and Mann-Whitney U test was used for two independent groups that were not normally distributed. Paired sample t-test was used if repeated measurements in dependent groups were normally distributed, and Wilcoxon test was used if they were not normally distributed. Kolmogorov-Smirnow normality test analysis was performed for normality analysis. Multivariate logistic regression analysis was used to determine the factors affecting the QTc interval. The results were evaluated at a 95% confidence interval, and the statistical significance level was defined as p <0.05. All analyses were performed using the IBM SPSS-25 (Statistical Package for Social Sciences, Chicago, Illinois, USA) package program.

## Results

In our study, 90 delirious patients were randomly assigned to receive either haloperidol or dexmedetomidine treatment in a 1-to-1 ratio. The patients had a mean age of 75.2±12.9 years, with half being female. The mean APACHE II score was 32.4±6.2, and the mean GCS was 9.2±1.8. The average ICU stay was 25.8±26.4 days, and the mean time to delirium onset was 142±173.8 hours. ICU mortality was 53.3% (48 patients). Following drug infusions, 55.6% of patients experienced at least a 20 ms QTc prolongation, while 31.1% had at least a 20 ms decrease in QTc intervals.

The demographic and clinical characteristics, including age, gender, body mass index (BMI), Acute Physiology and Chronic Health Evaluation II (APACHE II) score, and Glasgow Coma Scale (GCS) score, showed no statistically significant differences between the two groups (p=0.14, p=0.84, p=0.95, p=0.58, and p=0.48, respectively). In-hospital mortality rates were similar between the dexmedetomidine and haloperidol groups (48.9% vs. 57.8%, p=0.4). Before drug infusions, systolic blood pressure (SBP) and arterial oxygen saturation (SpO2) levels were lower in the dexmedetomidine group compared to the haloperidol group (SBP: 117.5±23.4 vs. 137.5±24.4, p<0.001; SpO2: 96.2±2.2 vs. 97.2±2.3, p=0.04, respectively). Parameters such as heart rate, Ramsay Sedation Scale score, and Richmond Agitation-Sedation Scale (RASS) score before drug infusions were similar between the two groups (p=0.83, p=0.26, p=0.18, respectively). There was no patient with drug addiction before intensive care admission, and no patient developed drug addiction during the treatment process. Additionally, the QT and corrected QT (QTc) intervals showed no significant differences between the dexmedetomidine and haloperidol groups before drug infusions (p=0.85, p=0.99, respectively). Table 1 provides a tabulated summary of the baseline characteristics based on the respective treatments with haloperidol and dexmedetomidine.

**Table 1:** Patient characteristics according to treatment groups.

**Patient characteristics**	**Dexmedetomidine (n=45)**	**Haloperidol (n=45)**	**P value**
Age (years) (mean±SD)	77.2±9.0	73.2±15.8	0.14
Sex (male%)	48.9	51.1	0.84
BMI (kg/m^2^) (mean±SD)	28.0±6.6	28.0±6.9	0.95
APACHE II (mean±SD)	32.8±5.3	32.0±7.0	0.58
GCS (mean±SD)	9.1±2.2	9.4±1.3	0.48
Hospitalization period (day) (median±SD)	16±13	21±34	0.016*
Time to delirium (hour) (median±SD)	48±197	120±146	0.27
Ventilator duration (day) (median±SD)	14±13.4	21±34.7	0.013*
Drug infusion time (hour) (median±SD)	108±95.2	126±75.5	0.94
Total amount of drug (mg) (median±SD)	3.8±1.6	150±107.4	<0.001*
In-hospital mortality (%)	48.9	57.8	0.4
Pre-infusion SBP (mmHg) (mean±SD)	117.5±23.4	137.5±24.4	<0.001*
Pre-infusion HR (bpm) (mean±SD)	98.5±25.6	97.5±19.7	0.83
Pre-infusion SpO2 (mean±SD)	96.2±2.2	97.2±2.3	0.04*
Pre-infusion Ramsay SS (median±SD)	1.3±0.8	1.5±1.0	0.26
Pre-infusion RASS score (median±SD)	2.45±1.45	1.96±2.2	0.18
Pre-infusion QT interval (ms) (mean±SD)	360.5±81.7	363.2±51.1	0.85
Pre-infusion QTc interval (ms) (mean±SD)	409.4±63.1	427.3±45.8	0.99

APACHE: Acute Physiology and Chronic Health Evaluation, BMI: Body mass index, bpm: Beats per minute, GCS: Glasgow Coma Score, HR: Heart rate, QTc: Corrected QT, RASS: Richmond Agitation-Sedation Scale, SBP: Systolic blood pressure, SD: Standard deviation, SpO2: Pulse oximeter oxygen saturation, SS: Sedation scale

**Fig. 1:**
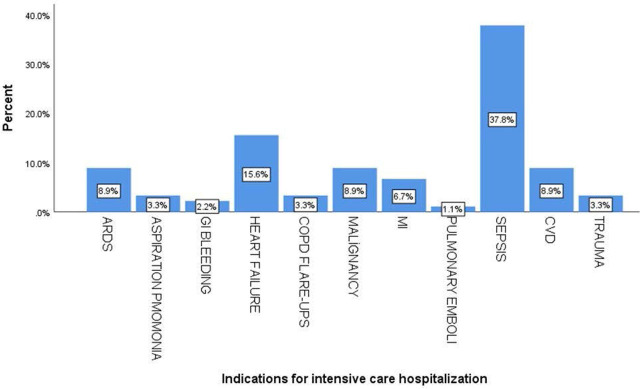
Indications for hospitalization in the intensive care unit.

The most common reasons for ICU hospitalization were sepsis (37.8%) and worsening heart failure (15.6%). Other indications for hospitalization are depicted in Figure 1. Patients were assigned to Haloperidol and Dexmedetomidine groups in a similar ratio according to their comorbidities. Medical treatments were arranged in consultation with the relevant branch physician during intensive care unit follow-up. During the administration of Haloperidol and Dexmedetomidine, other medical treatments were not given or their doses were not changed because additional effects reflected on ECG may occur. Therefore, additional ECG effects of Haloperidol and Dexmedetomidine were not compared in comorbidity groups individually.

**Fig. 2:**
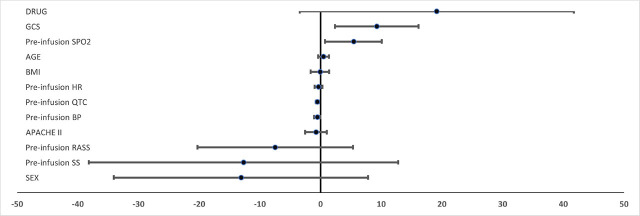
Forest plot graph of the relationship between baseline characteristics of patients and QTc prolongation after drug infusions.

Statistically significant concordant correlations were found between Glasgow Coma Scale (GCS) scores and arterial oxygen saturation (SpO2) levels with the extent of corrected QT (QTc) interval prolongation (p=0.02 and p=0.01, respectively). In contrast, a statistically significant discordant relationship was identified between pre-infusion QTc intervals and the degree of QTc prolongation following infusion (p=0.001). Notably, no significant associations were found between QTc prolongation and other relevant patient characteristics, as shown in Figure 2.

According to demographic data analyzed by gender, female patients were older and had lower GCS scores (age 78.2±13.3, 72.2±11.9, p= 0.027; GCS 8.8±2.2, 9.7±1.1, p=0.023, respectively). There was no gender difference in demographic and clinical data before drug infusion, except for age and GCS.

In the dexmedetomidine-administered group, a noteworthy inverse correlation was found between age and the duration of hospitalization, time to onset of delirium, pre-infusion Ramsay Sedation Scale score, as well as the maximal change observed in the corrected QT (QTc) interval post-infusion (correlation coefficient (r) = −0.464, p = 0.001; r = −0.438, p = 0.003; r = −0.338, p = 0.023; r = −0.338, p = 0.023, respectively). Conversely, a positive correlation emerged between age and Richmond Agitation-Sedation Scale (RASS) score, along with pre-infusion QT interval duration (r = 0.339, p = 0.023, and r = 0.319, p = 0.033, respectively). No statistically significant correlations were found between duration of ventilator support and pre-infusion QTc interval or age. In contrast, within the haloperidol-administered group, age did not show any significant correlations with other assessed factors.

Following standard clinical practice, patients were categorized based on their body mass index (BMI). Specifically, patients with a BMI of 30 or higher were classified as obese, while those with a BMI below 30 were categorized as non-obese. Patients were compared according to their obesity status regarding demographic and clinical data before infusion. Thirty percent (27 patients) of the patients were obese. Before drug infusions, SpO2 was lower in obese patients than in non-obese patients (97.1±2.2, 95.7±2.2, p=0.07, respectively). Other patient characteristics were similar in obese and non-obese patients.

After dexmedetomidine treatment, there was no significant change in SBP, heart rate, and Ramsay Sedation Scale (p=0.35, p=0.66, p=0.20, respectively), but a significant increase in SpO2 and a significant decrease in RASS were observed (SpO2: 96.2±2.2, 96.9±2.3, p=0.004; RASS: 2.5±1.5, 1.2±1.2, p<0.001). After haloperidol treatment, there was no significant change in SpO2 (p=0.59), but a significant decrease in SBP, heart rate, and RASS and a significant increase in Ramsay Sedation Scale were observed (SBP: 137.5±24.4, 117.4±26.0, p<0.001, heart rate: 97.5±19.7, 88.5±27.8, p=0.014, RASS: 2.0±2.2, 0.40±1.5, p<0.001, Ramsay Sedation Scale: 1.5±1.0, 1.8±1.0, p=0.015) (Table 2).

**Table 2.** Blood pressure, heart rate, oxygen saturation, sedation depth, and QT interval before and after haloperidol and dexmedetomidine treatment.

**Patient characteristics**	**Dexmedetomidine (n=45)**			**Haloperidol (n=45)**		

	**Pre-infusion**	**Post-infusion**	**P value**	**Pre-infusion**	**Post-infusion**	**P value**
Systolic blood pressure (mmHg)	117.6±3.4	113.2±27.2	0.345	137.5±24.4	117.4±26.0	<0.001*
Heart rate (bpm)	98.5±25.7	100.4±23.1	0.664	97.5±19.7	88.5±27.8	0.014*
Pulse oximeter oxygen saturation (SpO2) (%)	96.2±2.2	96.9±2.3	0.004*	97.2±2.3	97.0±2.2	0.585
Ramsay Sedation scale	1.3±0.8	1.4±0.6	0.204	1.5±1.0	1.8±1.0	0.015*
RASS	2.5±1.5	1.2±1.2	<0.001*	2.0±2.2	0.40±1.5	<0.001*

bpm: beats per minute, RASS: Richmond Agitation and Sedation Scale

**Fig. 3.**
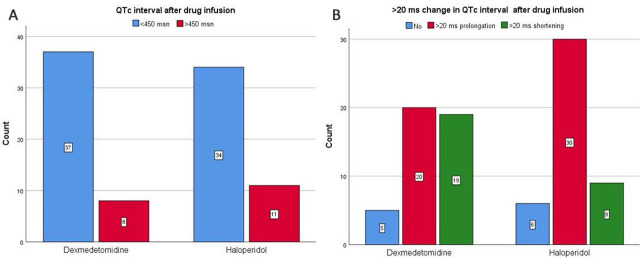
A Patients with QTc interval above 450 msec after drug infusions, B: Patients with >20 msec prolongation in QTc interval after drug infusions.

The QTc interval was above 450 ms after drug infusions in 17.8% (8) of patients receiving dexmedetomidine treatment and 24.4% (11 patients) of patients receiving haloperidol treatment, with no significant difference between the groups (p=0.6). The proportion of patients with a >20 ms increase in QTc interval after drug infusion was 44.4% (20 patients) in patients receiving dexmedetomidine treatment, 66.7% (30 patients) in patients receiving haloperidol treatment, with a significant difference between the groups (p=0.06). The proportion of patients with a >20 ms shortening in QTc interval after drug infusion was 43.2% (19 patients) in patients receiving dexmedetomidine treatment, 20.0% (9 patients) in patients receiving haloperidol treatment, with a significant difference between the groups (p=0.05) (Figure 3).

**Fig. 4:**
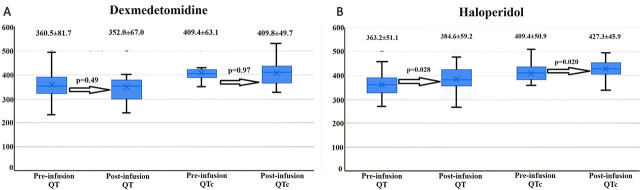
The effect of dexmedetomidine (A) and haloperidol (B) treatment on QT and QTc interval in box plot graph.

There was no significant change in QT and QTc interval after dexmedetomidine treatment (QT: 360.5±81.7, 352.0±67.0, p= 0.491; QTc: 409.4±63.1, 409.8±49.7, p=0.974). However, a significant increase was observed in both QT and QTc intervals after haloperidol treatment (QT: 363.2±51.1, 384.6±59.2, p=0.028; QTc: 409.4±50.9, 427.3±45.9, p=0.020*) (Figure 4).

## Discussion

The findings of this prospective randomized clinical study provide important insights into the effects of haloperidol and dexmedetomidine on various clinical outcomes in ICU patients. The study showed that haloperidol resulted in a significant increase in both QT and QTc intervals, which could have implications for cardiac function and patient outcomes. This finding is consistent with previous studies that have shown that haloperidol can have adverse effects on cardiac repolarization and increase the risk of cardiac arrhythmias [22,23,24,25,26].

In contrast, dexmedetomidine did not result in a significant change in either QT or QTc intervals, suggesting that this medication may be a safer alternative for ICU patients who require sedation. Additionally, the study found that dexmedetomidine was associated with a shorter duration of mechanical ventilation and a shorter length of hospital stay, which could have important implications for improving patient outcomes and reducing healthcare costs.

Overall, the study highlights the importance of carefully weighing the risks and benefits of different medications in the ICU setting, particularly when it comes to sedation and management of delirium. The findings suggest that dexmedetomidine may be a preferable alternative to haloperidol for ICU patients who require sedation, as it appears to have fewer adverse effects on cardiac function and may be associated with improved clinical outcomes.

In previous studies, dexmedetomidine and haloperidol were not compared in terms of their ECG properties. Initial concerns regarding the potential of haloperidol to prolong the QT interval emerged from several case reports and small case series documenting occurrences of Torsades de Pointes (TdP) and significant QT interval prolongation during intravenous haloperidol therapy [22]. Although these early reports provided initial evidence, they were limited in scope and not sufficient to definitively establish the risk. Consequently, cardiac monitoring was recommended when administering intravenous haloperidol [22].

Subsequent larger studies have further explored this relationship. Perrault et al. observed a 2% incidence of delirium during intensive care unit follow-ups after coronary bypass grafting surgery, noting the potential for TdP development even in the absence of marked QT prolongation following haloperidol treatment [23–24]. These findings have been corroborated by additional research indicating that the risk of TdP can occur not only with high doses but also at relatively low oral doses of haloperidol [25,26]. Thus, while initial evidence was limited, more recent and extensive studies have reinforced the importance of vigilant cardiac monitoring to mitigate the risk of TdP in patients receiving haloperidol.

Dexmedetomidine has been added to the list of drugs possibly prolonging QTc based on pediatric patient case reports [27]. However, there is no published article on the use of dexmedetomidine in causing TdP. A study also reports that dexmedetomidine shortens the QT interval after a bolus dose [28]. Although dexmedetomidine has no direct effect on QTc, dexmedetomidine-induced bradycardia indirectly triggers congenital long QT syndrome is the most likely explanation [29].

Our study did not observe dexmedetomidine-related QTc prolongation and torsade de pointes. Haloperidol treatment was associated with a significant prolongation of both QT and QTc, but no malignant cardiac arrhythmia existed. Dexmedetomidine treatment was found to be safer compared to haloperidol treatment in terms of the relationship to QT interval.

Low GCS and SpO2 are associated with poor prognosis. One study found that low GSC and hypoxia were associated with prolongation in the QTc interval [30]. In our study, the increase in QTc interval was higher after drug administration in patients with higher GCS and SpO2 levels before drug administration. This result may be due to higher doses of sedative drugs given to patients with high GCS and SpO2 levels.

The APACHE II score is used to measure the severity of illness and predict mortality risk in ICU patients. A Study has indicated that higher APACHE II scores, which reflect greater illness severity, are associated with prolonged QT intervals [31]. This relationship can be due to various factors, including the effects of critical illness on the cardiovascular system, electrolyte imbalances, and the use of medications that can prolong the QT interval. Understanding this relationship is important for managing ICU patients, as those with higher APACHE II scores may require more careful monitoring of their QT intervals to prevent adverse cardiac events. In our study, a relationship between QT interval and APACHE II score may not have been detected due to reasons such as paying attention to electrolyte imbalance and carefully monitoring comorbidities in consultation with the relevant branch judge.

The relationship between the QT interval and the Ramsay Sedation Scale is not as well established as the relationship between the QT interval and the APACHE II score. While there is no direct established relationship between the QT interval and the Ramsay Sedation Scale score, the factors associated with sedation and critical illness management can indirectly affect the QT interval. Thus, careful monitoring of the QT interval is warranted in sedated patients, especially those receiving medications known to prolong the QT interval. In our study, there was no significant change in the Ramsay Sedation Scale score after dexmedetomidine treatment, whereas there was a significant increase in the Ramsay Sedation Scale score after haloperidol treatment. This result suggests that the significant increase in the depth of sedation may also have an effect on the effect of haloperidol on the QT interval.

The Richmond Agitation-Sedation Scale is a tool used to assess the level of sedation and agitation in patients, particularly in the ICU. While there is no direct, well-documented relationship between RASS score and the QT interval, the factors associated with sedation, agitation, and critical illness management can indirectly influence the QT interval. Careful monitoring of the QT interval is important in patients with extreme RASS scores, especially those receiving medications known to affect the QT interval. In our study, there was a decrease in RASS score in patients receiving both dexmedetomidine and haloperidol treatment. Due to this one-way change in the RASS score, it may have caused no relationship between this score and the QT interval.

The risk between an increase in the QTc interval and the development of malignant arrhythmia is known [32]. The effect of the initial QT interval on QT prolongation has not been investigated. Our study found that numerical prolongation of the QT interval was more prominent with drugs in patients with shorter QT intervals.

An inverse relationship was found between maximum QTc interval prolongation and age, time to delirium, hospitalization time, and lower Ramsay sedation scale in patients receiving dexmedetomidine treatment. However, this relationship was not observed in haloperidol treatment. A concordant relationship between age and QTc is expected [33]. However, as previously explained, the requirement for higher doses of sedative drugs in younger, agitated, and early delirium patients should have prolonged the QTc interval more significantly.

The most common cardiovascular adverse effects of haloperidol are tachycardia and hypotension or hypertension [13]. The most common cardiovascular adverse effects of dexmedetomidine are bradycardia, atrial fibrillation, hypoxia, and hypotension or hypertension [19,20,30]. Our study observed no significant effect of dexmedetomidine on cardiovascular outcomes such as SBP and heart rate. However, haloperidol caused significant reductions in SBP and heart rate.

Our study contributes significantly to the existing literature by directly comparing the effects of haloperidol and dexmedetomidine on QTc interval prolongation in ICU patients. While previous studies have explored the individual impacts of these medications on cardiac function and arrhythmias, few have conducted a head-to-head comparison in an ICU setting. This direct comparison allows for a more nuanced understanding of the differential effects of these commonly used sedatives, particularly concerning their potential to prolong QTc intervals and influence patient outcomes. Additionally, our study sheds light on the safety profile of dexmedetomidine compared to haloperidol, providing valuable insights for clinicians in selecting appropriate sedation strategies for ICU patients with delirium. These findings underscore the importance of tailored pharmacological approaches in critical care settings and highlight the need for further research to elucidate the mechanisms underlying these drug-specific effects on cardiac repolarization.

### Limitations

There were significant limitations in our study. First, although our study was randomized and prospective, the small number of patients and the single-center nature of the study were important limitations. Another limitation is that propensity score matching was not performed due to the small number of patients. Finally, patients’ QTc distances were measured only according to Bazett’s formula, and no adjustment was made for age and gender. Adding one or more of the Fridericia, Hodges, and Framingham formulas to the analysis could increase the reliability of the study results.

## Conclusion

The results of our study demonstrated a significant increase in QTc interval in critically ill patients treated with haloperidol, in contrast to those treated with dexmedetomidine, where no significant change in QTc interval was observed. It is noteworthy that the mean QTc increase and length of QTc interval were higher in patients treated with haloperidol.

Our findings have important clinical implications, indicating that dexmedetomidine may be a safer alternative to haloperidol for sedation in critically ill patients, especially those with pre-existing cardiovascular disease. Our study highlights the need for careful consideration of the choice of sedation medication in critically ill patients, particularly those with pre-existing cardiovascular disease, to minimize the risk of adverse cardiac events. Further studies are required to validate our findings and establish the optimal sedation strategy for critically ill patients with cardiovascular disease.

## Highlights

– The study underscores dexmedetomidine as a safer option for sedation in critically ill patients, avoiding significant QTc interval changes linked to haloperidol use.
– Haloperidol administration in ICU patients raises cardiac concerns due to notable QTc interval prolongation, warranting caution.
– Dexmedetomidine emerges as a potential boon for ICU management, potentially reducing ventilation duration and hospital stays, thus improving critical care patient outcomes.
